# A review of some techniques for inclusion of domain-knowledge into deep neural networks

**DOI:** 10.1038/s41598-021-04590-0

**Published:** 2022-01-20

**Authors:** Tirtharaj Dash, Sharad Chitlangia, Aditya Ahuja, Ashwin Srinivasan

**Affiliations:** 1 Department of Computer Science and Information Systems, Anuradha and Prashanth Palakurthi Centre for AI Research (APPCAIR), BITS Pilani, K.K. Birla Goa Campus, Goa, 403726 India; 2 Department of Electrical and Electronics Engineering, Anuradha and Prashanth Palakurthi Centre for AI Research (APPCAIR), BITS Pilani, K.K. Birla Goa Campus, Goa, 403726 India

**Keywords:** Engineering, Computer science, Information technology

## Abstract

We present a survey of ways in which existing scientific knowledge are included when constructing models with neural networks. The inclusion of domain-knowledge is of special interest not just to constructing scientific assistants, but also, many other areas that involve understanding data using human-machine collaboration. In many such instances, machine-based model construction may benefit significantly from being provided with human-knowledge of the domain encoded in a sufficiently precise form. This paper examines the inclusion of domain-knowledge by means of changes to: the input, the loss-function, and the architecture of deep networks. The categorisation is for ease of exposition: in practice we expect a combination of such changes will be employed. In each category, we describe techniques that have been shown to yield significant changes in the performance of deep neural networks.

## Introduction

**Figure 1:**
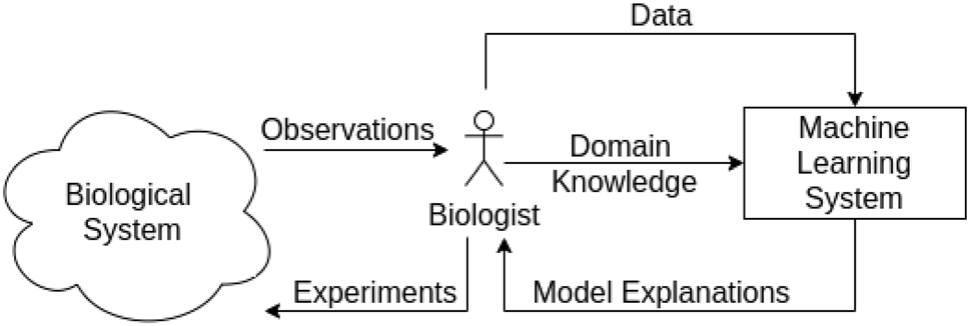
An example of AI for Science. The human-in-the-loop is a biologist. The biologist conducts experiments in a biological system, obtains experimental observations. The biologist then extracts data that can be used to construct machine learning model(s). Additionally, the machine learning system has access to domain knowledge that can be obtained from the biologist. The machine learning system then conveys its explanations to the biologist.

**Figure 2:**
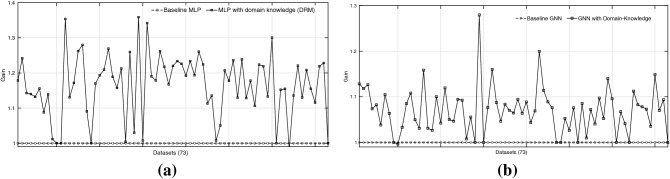
The plots from^6^ showing gains in predictive accuracy of (**a**) multilayer perceptron (MLP), and (**b**) graph neural network (GNN) with the inclusion of domain-knowledge. The domain knowledge inclusion method in (**a**) is a simple technique known as ‘propositionalisation’ ^7^; and, the method in (**b**) is a general technique of incorporating domain-knowledge using bottom-graph construction. The results shown are over 70 datasets. No importance to be given to the line joining two points; this is done for visualisation purpose only.

Science is a cumulative enterprise, with generations of scientists discovering, refining, correcting and ultimately increasing our knowledge of how things are. The accelerating pace of development in software and hardware for machine learning–in particular, the area of deep neural networks (DNNs)–inevitably raises the prospect of Artificial Intelligence for Science^1^. That is, how can we best use AI methods to accelerate our understanding of the natural world? While ambitious plans exist for completely automated AI-based robot scientists^2^, the immediately useful prospect of using AI for Science remains semi-automated. An example of such a collaborative system is in Fig. 1. For such systems to work effectively, we need at least the following: (1) We have to be able to tell the machine what we know, in a suitably precise form; and (2) The machine has to be able to tell us what it has found, in a suitably understandable form. While the remarkable recent successes of deep neural networks on a wide variety of tasks makes a substantial case for their use in model construction, it is not immediately obvious how either (1) or (2) should be done with deep neural networks. In this paper, we examine ways of achieving (1), that is, the techniques for constructing deep neural networks from data and domain-knowledge concerning the problem. Understanding models constructed by deep neural networks is an area of intense research activity, and good summaries exist elsewhere^3,4^. To motivate the utility of providing domain-knowledge to a deep network, we reproduce two results from^5^ in Fig. 2, which shows that predictive performance can increase significantly, even with a simplified encoding of domain-knowledge (see Fig. 2a).

It is unsurprising that a recent report on AI for Science^1^ identifies the incorporation of domain-knowledge as one of the 3 Grand Challenges in developing AI systems:

“ML and AI are generally domain-agnostic...Off-the-shelf [ML and AI] practice treats [each of these] datasets in the same way and ignores domain knowledge that extends far beyond the raw data...Improving our ability to systematically incorporate diverse forms of domain knowledge can impact every aspect of AI.”

**Figure 3:**
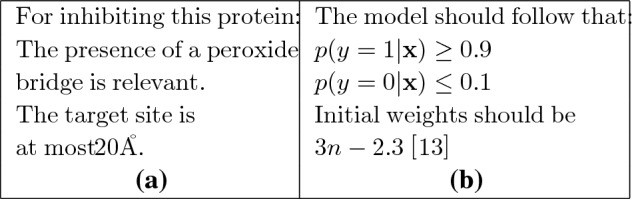
Informal descriptions of (**a**) logical; and (**b**) numerical constraints.

But it is not just the construction of scientific-assistants that can benefit from this form of man-machine collaboration, and “human-in-the-loop” AI systems are likely to play an increasingly important role in engineering, medicine, healthcare, agriculture, environment and so on^8^. In this survey, we restrict the studies on incorporation of domain-knowledge into neural networks, with 1 or more hidden layers. If the domain-knowledge expressed in a symbolic form (for example, logical relations that are known to hold in the domain), then the broad area of hybrid neural-symbolic systems (see for example,^9,10^) is clearly relevant to the material in this paper. However, the motivation driving the development of hybrid systems is much broader than this paper, being concerned with general-purpose neural-based architectures for logical representation and inference. Here our goals are more modest: we are looking at the inclusion of problem-specific information into machine-learning models of a kind that will be described shortly. We refer the reader to^11^ for reviews of work in the broader area of neural-symbolic modelling. More directly related to this paper is the work on “informed machine learning”, reviewed in^12^. We share with this work the interest in prior knowledge as an important source of information that can augment existing data. However, the goals of that paper are more ambitious than here. It aims to identify categories of prior knowledge, using as dimensions: the source of the knowledge, its representation, and its point of use in a machine-learning algorithm. In this survey, we are only concerned with some of these categories. Specifically, in terms of the categories in^12^, we are interested in implicit or explicit sources of domain-knowledge, represented either as logical or numeric constraints, and used at the model-construction stage by DNNs. Informal examples of what we mean by logical and numerical constraints are shown in Fig. 3. In general, we will assume logical constraints can, in principle, be represented as statements in propositional logic or predicate logic. Numerical constraints will be representable, in principle, as terms in an objective function being minimised (or maximised), or prior distributions on models. We believe this covers a wide range of potential applications, including those concerned with scientific discovery.

### Focus of the paper

**Figure 4:**
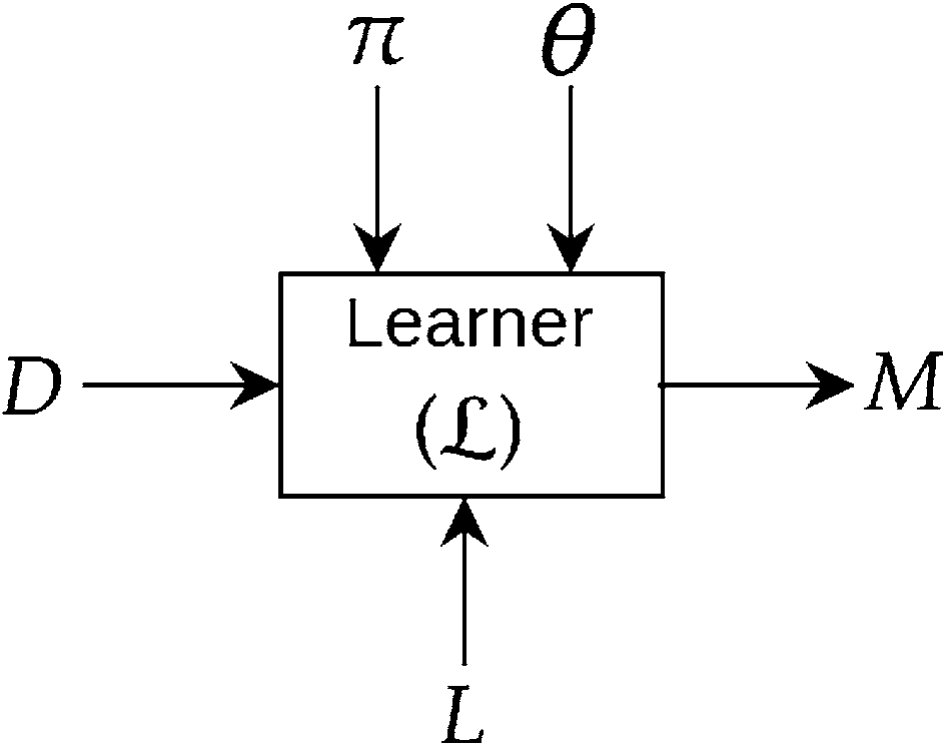
Construction of a deep model *M* from data (*D*) using a learner (${\mathcal {L}}$). We use $\pi$ to denote the structure (organisation of various layers, their interconnections etc.) and $\varvec{\theta }$ to denote the parameters (synaptic weights) of the deep network. *L* denotes the loss function (for example, cross-entropy loss in case of classification).

We adhere to the following informal specification for constructing a deep neural network: given some data *D*, a structure and parameters of a deep network (denoted by $\pi$ and $\varvec{\theta }$, respectively), a learner ${\mathcal {L}}$ attempts to construct a neural network model *M* that minimises some loss function *L*. Fig. 4 shows a diagrammatic representation. Note that: (a) we do not describe how the learner ${\mathcal {L}}$ constructs a model *M* given the inputs. But, it would be normal for the learner to optimise the loss *L* by performing an iterative estimation of the parameters $\theta$, given the model structure $\pi$; and (b) we are not concerned with how the constructed deep model *M* will be used. However, it suffices to say that when used, the model *M* would be given one or more data-instances encoded in the same way as was provided for model-construction.

**Figure 5:**
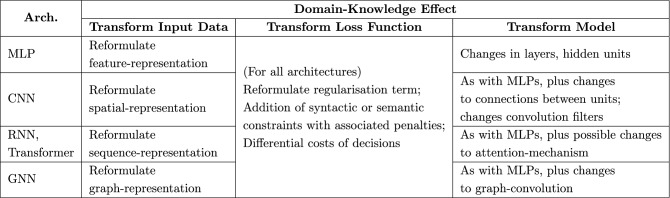
Some implications of using domain-knowledge for commonly-used deep network architectures. Although attention-mechanism has also been used recently in many deep network architectures, we mention it only for RNNs and transformers as it is more prominently being used for sequence learning.

In the literature, domain knowledge–also called background knowledge–does not appear to have an accepted definition, other than that, it refers to information about the problem. This information can be in the form of relevant features, concepts, taxonomies, rules-of-thumb, logical constraints, probability distributions, mathematical distributions, causal connections and so on. In this paper, we use the term “domain-knowledge” to refer to problem-specific information that can directly be translated into alterations to the principal inputs of Fig. 4. That is, by domain-knowledge we will mean problem-specific information that can change: (1) The input data to a deep network; (2) The loss-function used; and (3) The model (that is, the structure or parameters) of the deep network. In a sense, this progression reflects a graded increase in the complexity of changes involved. Figure 5 tabulates the principal implications of this position for commonly-used deep learning architectures.

The rest of the paper is organised as follows: Section 2 describes inclusion of domain-knowledge by augmenting or transformation of inputs; Section 3 describes changes that have been employed to loss-functions; and Section 4 describes biases on parameters and changes to the structure of deep networks. Section 5 outlines some major challenges related to the inclusion of domain-knowledge in the ways we describe. In this section, we also present perspectives on the relevance of the use of domain-knowledge to aspects of Responsible AI, including ethics, fairness, and explainability of DNNs.

### Transforming the input data

**Figure 6:**
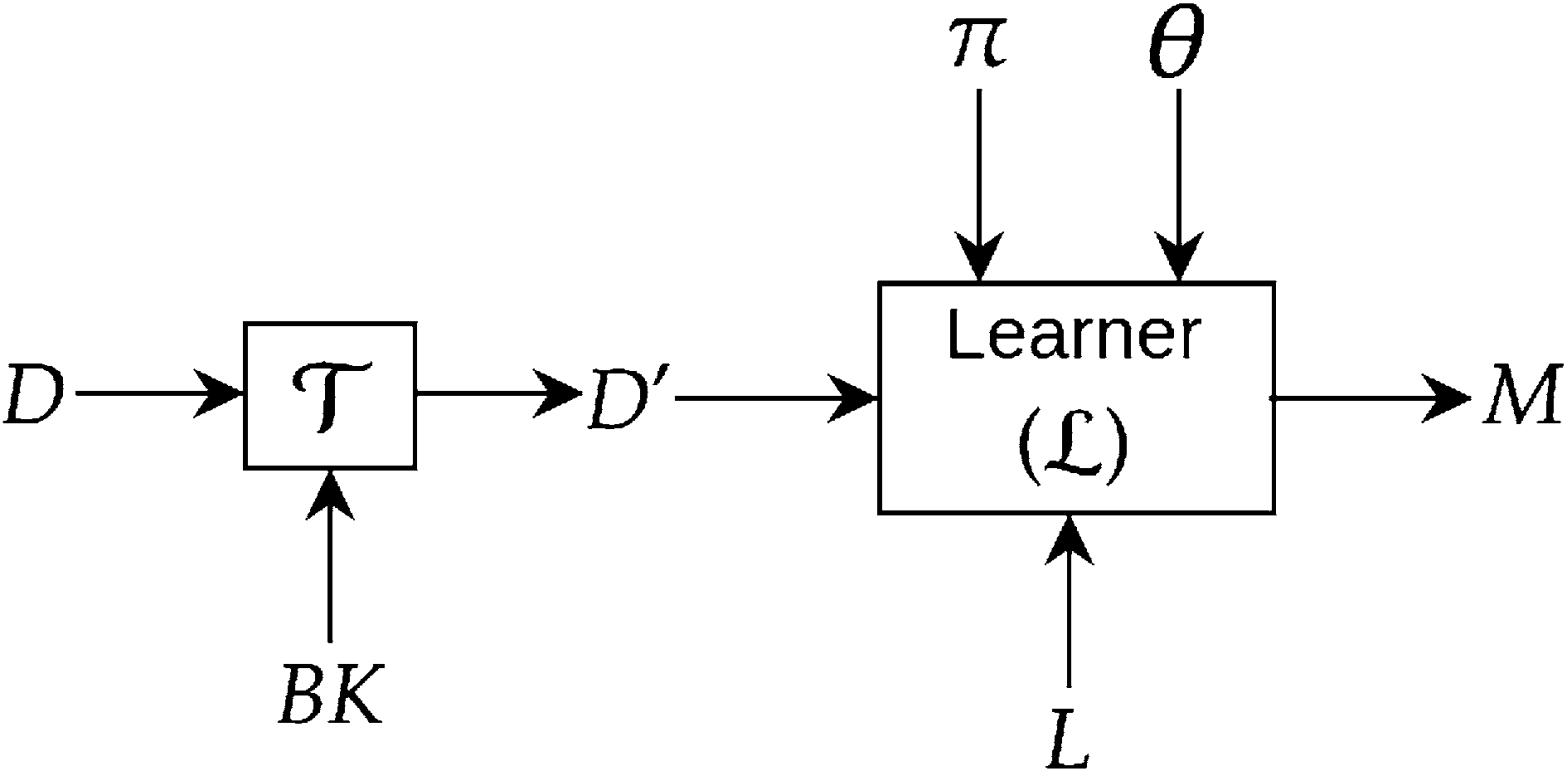
Introducing background knowledge into deep network by transforming data. ${\mathcal {T}}$ is a transformation block that takes input data *D*, background knowledge (*BK*) and outputs transformed data $D'$ that is then used to construct a deep model using a learner ${\mathcal {L}}$.

One of the prominent approaches to incorporate domain-knowledge into a deep network is by changing inputs to the network. Here, the domain-knowledge is primarily in symbolic form. The idea is simple: If a data instance could be described using a set of attributes that not only includes the raw feature-values but also includes more details from the domain, then a standard deep network could then be constructed from these new features. A simple block diagram in Fig. 6 shows how domain knowledge is introduced into the network via changes in inputs. In this survey, we discuss broadly two different ways of doing this: (a) using relational features, mostly constructed by a method called propositionalisation^7^ using another machine learning system (for example, Inductive Logic Programming) that deals with data and background knowledge; (b) without propositionalisation.

### Propositionalisation

The pre-eminent form of symbolic machine learning based on the use of relations in first-order logic is Inductive Logic Programming (ILP)^14^, which has an explicit role for domain-knowledge being incorporated into learning. The simplest use of ILP^14^ to incorporate *n*-ary relations in domain knowledge into a neural network relies on techniques that automatically “flatten” the domain-knowledge into a set of domain-specific relational features. Although not all DNNs require data to be a set of feature-vectors, this form of data representation is long-standing and still sufficiently prevalent. In logical terms, we categorise feature-based representations as being encodings in propositional logic. The reader would point out, correctly, that feature-values may not be Boolean. This is correct, but we can represent non-Boolean features by Boolean-valued propositions (for example, a real-valued feature *f* with value 4.2 would be represented by a corresponding Boolean feature $f'$ that has the value 1 if $f=4.2$ and 0 otherwise). With the caveat of this rephrasing, it has of course been possible to provide domain-knowledge to neural networks by employing domain-specific features devised by an expert. However, we focus here on ways in which domain-knowledge encoded as rules in propositional and first-order logic has been used to construct the input features for a deep neural network. Techniques for automatic construction of Boolean-valued features from relational domain-knowledge have a long history in the field of ILP^15–17^, originating from the LINUS^7^. Often called *propositionalisation*, the approach involves the construction of features that identify the conditions under which they take on the value 1 (or 0). For example, given (amongst other things) the definition of benzene rings and of fused rings, an ILP-based propositionalisation may construct the Boolean-valued feature that has the value 1 if a molecule has 3 fused benzene rings, and 0 otherwise. The values of such Boolean-valued features allows us to represent a data instance (like a molecule) as a Boolean-valued feature-vector, which can then be provided to a neural network. There is a long history of propositionalisation: see^18^ for a review of some of early use of this technique, and^19,20^ who examine the links between propositionalisation and modern-day use of embeddings in deep neural networks. More clearly, the authors examine that both propositionalisation and embedding approaches aim at transforming data into tabular data format, while they are being used in different problem settings and contexts. One recent example of embedding is demonstrated in^21^ where the authors use different text-embedding approaches such as sentence encoder^22^ and GPT2^23^ to transform textual domain-knowledge into embedding vectors.

A direct application of propositionalisation, demonstrating its utility for deep networks has been its use in Deep Relational Machines (DRMs)^24^. A DRM is a deep fully-connected neural network with Boolean-valued inputs obtained from propositionalisation by an ILP engine. In^25^, Boolean-valued features from an ILP engine are sampled from a large space of possible relational features. The sampling technique is refined further in^26^.

The idea of propositionalisation also forms the foundation for a method known as ‘Bottom Clause Propositionalisation (BCP)’ to propositionalise the literals of a most-specific clause, or “bottom-clause” in ILP. Given a data instance, the bottom-clause is the most-specific first-order clause that entails the data instance, given some domain-knowledge. Loosely speaking, the most-specific clause can be thought of “enriching” the data instance with all domain relations that are true, given the data instance. The construction of such most-specific clauses and their subsequent use in ILP was introduced in^27^. CILP++^28^ uses bottom-clauses for data instances to construct feature-vectors for neural networks. This is an extension to CIL$^2$P in which the neural network uses recurrent connections to enforce the background-knowledge during the training^29^.

Propositionalisation has conceptual and practical limitations. Conceptually, there is no variable-sharing between two or more first-order logic features^25^. That is, all useful compositions have to be pre-specified. Practically, this makes the space of possible features extremely large: this has meant that the feature-selection has usually been done separately from the construction of the neural network. In this context, another work that does not employ either propositionalisation or network augmentation considers a combination of symbolic knowledge represented in first-order logic with matrix factorization techniques^30^. This exploits dependencies between textual patterns to generalise to new relations.

Recent work on neural-guided program synthesis also explicitly includes domain-specific relations. Here programs attempt to construct automatically compositions of functional primitives. The primitives are represented as fragments of functional programs that are expected to be relevant. An example of neural-guided program synthesis that uses such domain-primitives is DreamCoder^31,32^. DreamCoder receives as inputs, the partial specification of a function in the form of some inputs–output pairs, and a set of low-level primitives represented in a declarative language. Higher-level domain-concepts are then abduced as compositions of these primitives via a neurally-guided search procedure based on a version of the Bayesian “wake-sleep” algorithm^33^. The deep networks use a (multi-hot) Boolean-vector encoding to represent functional compositions (a binary digit is associated with each primitive function, and takes the value 1 if and only if the primitive is used in the composite function).

There are methods that do not use an explicit propositionalisation step, but nevertheless amount to re-formulating the input feature-representation. In the area of “domain-adaptation”^34^, “source” problems act as a proxy for domain-knowledge for a related “target” problem (Superficially, this is also the setting underlying *transfer* learning. However, the principal difference is that source and target problems are closely related in domain-adaptation, but this need not be the case with transfer-learning. Transfer-learning also usually involves changes in both model-parameters and model-structure. Domain-adaptation does not change the model-structure: we consider these points in a later section). There is a form of domain-adaptation in which the target’s input representation is changed based on the source model. In^35^, for example, a feature-encoder ensures that the feature representation for the target domain that is the same as the one used for the source.

### Binary and *n*-ary relations

An influential form of representing relational domain-knowledge takes the form *knowledge graph*, which are labelled graphs, with vertices representing entities and edges representing binary relations between entities. A knowledge graph provides a structured representation for knowledge that is accessible to both humans and machines^36^. Knowledge graphs have been used successfully in variety of problems arising in information processing domains such as search, recommendation, summarisation^37^. Sometimes the formal semantics of knowledge graphs such as domain ontologies are used as sources for external domain-knowledge^38^. We refer the reader to^39^ to a comprehensive survey of this form of representation for domain-knowledge.

Incorporation of the information in a knowledge-graph into deep neural models–termed “knowledge-infused learning”–is described in^40,41^. This aims to incorporate binary relations contained in application-independent sources (like DBPedia, Yago, WikiData) and application-specific sources (like SNOMED-CT, DataMed). The work examines techniques for incorporating relations at various layers of deep-networks (the authors categorise these as “shallow”, “semi-deep” and “deep” infusion). In the case of shallow infusion, both the external knowledge and the method of knowledge infusion are shallow, utilising syntactic and lexical knowledge in word embedding models. In semi-deep infusion, external knowledge is involved through attention mechanisms or learnable knowledge constraints acting as a sentinel to guide model learning. Deep infusion employs a stratified representation of knowledge representing different levels of abstractions in different layers of a deep learning model to transfer the knowledge that aligns with the corresponding layer in the learning process. Fusing the information in a knowledge-graph in this way into various level of hidden representations in a deep network could also allow quantitative and qualitative assessment of its functioning, leading to knowledge-infused interpretability^42^.

There have been some recent advances in introducing external domain-knowledge into deep sequence models. For instance, in^38^, the authors incorporate domain-specific knowledge into the popular deep learning framework, BERT^43^ via a declarative knowledge source like drug-abuse ontology. The model constructed here, called Gated-K-BERT, is used for jointly extracting entities and their relationships from tweets by introducing the domain-knowledge using an entity position-aware module into the primary BERT architecture. The experimental results demonstrate that incorporating domain-knowledge in this manner leads to better relation extraction as compared to the state-of-the-art. This work could fall within the category of semi-deep infusion as described in^40,44^, in their study on learning from electronic health records show that the adjacency information in a medical knowledge graph can be used to model the attention mechanism in an LSTM-based RNN with attention. Whenever the RNN gets an entity (a medical event) as an input, the corresponding sub-graph in the medical knowledge graph (consisting of relations such as *causes* and *is-caused-by*) is then used to compute an attention score. This method of incorporating the medical relations into the RNN falls under the category of semi-deep knowledge infusion. While the above methods use the relational knowledge from a knowledge-graph by altering or adding an attention module within the deep sequence model, a recent method called KRISP^45^ introduces such external knowledge at the output (prediction) layer of BERT. This work could be considered under the category of shallow infusion of domain-knowledge as characterised by^40^.

Knowledge graphs can be used directly by specialised deep network models that can handle graph-based data as input (graph neural networks, or GNNs). The computational machinery available in GNN then aggregates and combines the information available in the knowledge graph (an example of this kind of aggregation and pooling of relational information is in^46^). The final collected information from this computation could be used for further predictions. Some recent works are in^47,48^, where a GNN is used for estimation of node importance in a knowledge-graph. The intuition is that the nodes (in a problem involving recommender systems, as in^48^, a node represents an entity) in the knowledge-graph can be represented with an aggregated neighbourhood information with bias while adopting the central idea of aggregate-and-combine in GNNs. The idea of encoding a knowledge graph directly for a GNN is also used in Knowledge-Based Recommender Dialog (KBRD) framework developed for recommender systems^49^. In this work, the authors treat an external knowledge graph, such as DBpedia^50^, as a source of domain-knowledge allowing entities to be enriched with this knowledge. The authors found that the introduction of such knowledge in the form of a knowledge-graph can strengthen the recommendation performance significantly and can enhance the consistency and diversity of the generated dialogues. In KRISP^45^, a knowledge-graph is treated as input for a GNN where each node of the graph network corresponds to one specific domain-concept in the knowledge graph. This idea is a consequence of how a GNN operates: it can form more complex domain-concepts by propagating information of the basic domain-concepts along the edges in the knowledge-graph. Further, the authors allow the network parameters to be shared across the domain-concepts with a hope to achieve better generalisation. We note that while knowledge-graph provide an explicit representation of domain-knowledge in the data, some problems contain domain-knowledge implicitly through an inherent topological structure (like a communication network). Clearly, GNNs can accommodate such topological structure just in the same manner as any other form of graph-based relations (see for example:^51^).

Going beyond binary relations in knowledge-graphs and treating *n*-ary relations as hyperedges, a technique called *vertex enrichment* is proposed in^5^. Vertex-enrichment is a simplified approach for the inclusion of domain-knowledge into standard graph neural networks (GNNs). The approach incorporates first-order background relations by augmenting the features associated with the nodes of a graph provided to a GNN. The results reported in^5^ show significant improvements in the predictive accuracy of GNNs across a large number datasets. The simplification used in vertex-enrichment has been made unnecessary in a recent proposal for transforming the most-specific clause constructed by ILP systems employing mode-directed inverse entailment (MDIE^27^). The transformation converts this clause into a graph can be directly used by any standard GNN^6^. Specifically, the transformation results in a labelled bipartite graph consisting of vertices representing predicates (including domain predicates) and ground terms. This approach reports better predictive performance than those reported in^5^, and includes knowledge-graphs as a special case. Most recently, this method has been combined successfully with deep generative sequence models for generating target-specific molecules, which demonstrates yet another real-world use-case of incorporating domain knowledge into deep networks^52^.

## Transforming the loss function

**Figure 7:**
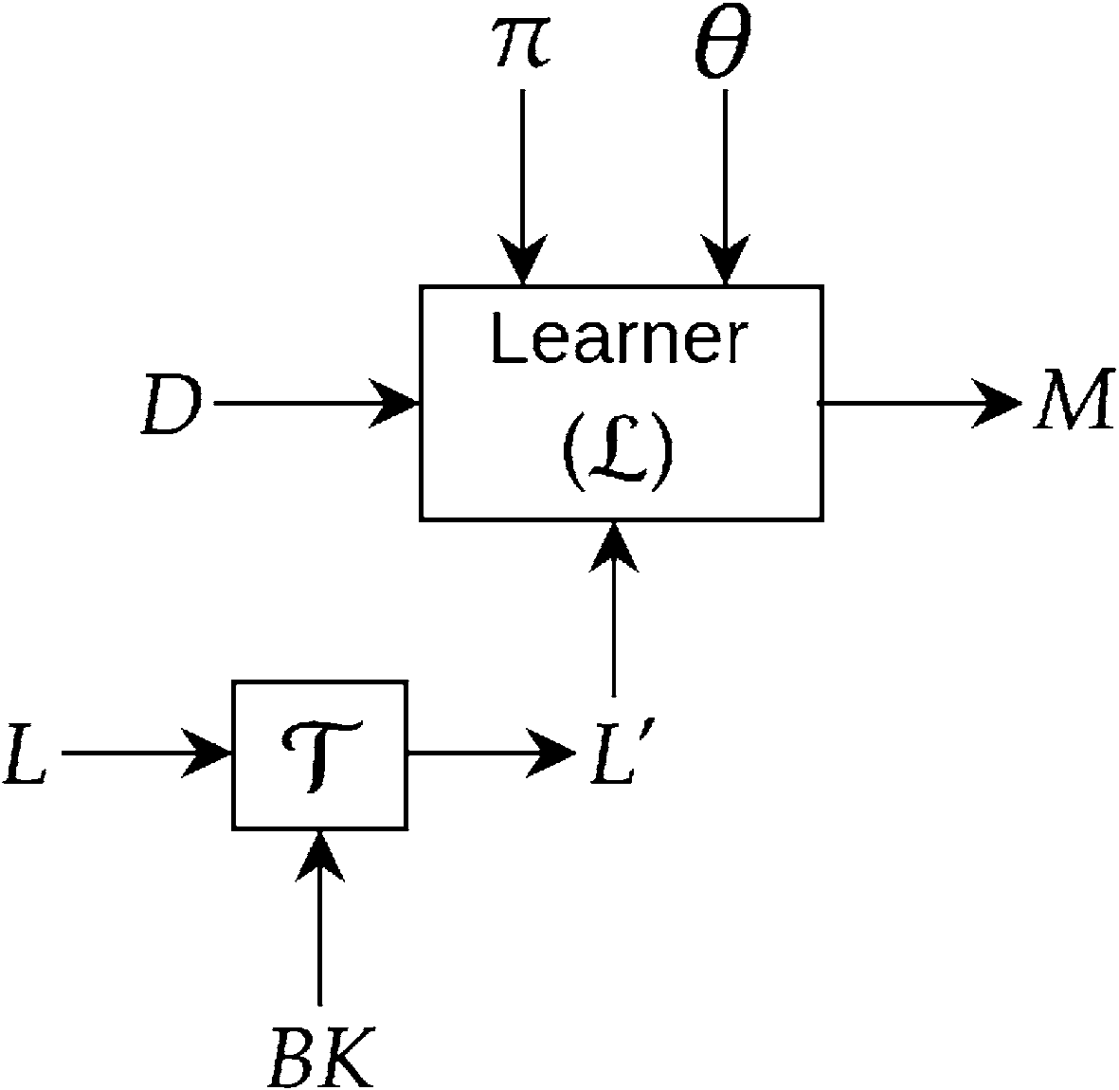
Introducing background knowledge into deep network by transforming the loss function *L*. ${\mathcal {T}}$ block takes an input loss *L* and outputs a new loss function $L'$ by transforming (augmenting or modifying) *L* based on background knowledge (*BK*). The learner ${\mathcal {L}}$ then constructs a deep model using the original data *D* and the new loss function $L'$.

One standard way of incorporating domain-knowledge into a deep network is by introducing “penalty” terms into the loss (or utility) function that reflect constraints imposed by domain-knowledge. The optimiser used for model-construction then minimises the overall loss that includes the penalty terms. Fig. 7 shows a simple block diagram where a new loss term is introduced based on the background knowledge. We distinguish two kinds of domain constraints–syntactic and semantic–and describe how these have been used to introduce penalty terms into the loss function.

### Syntactic Loss

The usual mechanism for introducing syntactic constraints is to introduce one or more *regularisation* terms into the loss function. The most common form of regularisation introduces penalties based on model complexity (number of hidden layers, or number of parameters and so on: see, for example,^53^).

A more elaborate form of syntactic constraints involves the concept of *embeddings*. Embeddings refer to the relatively low-dimensional learned continuous vector representations of discrete variables. Penalty terms based on “regularising embeddings” are used to encode syntactic constraints on the complexity of embeddings.^54^ was an early work in this direction, in which the authors proposed a strategy to establish constraints by designating each node in a Hopfield Net to represent a concept and edges to represent their relationships and learn these nets by finding the solution which maximises the greatest number of these constraints.^55^ was perhaps the first method of regularising embeddings from declarative knowledge encoded in first-order logic. The proposal here is for mapping between logical statements and their embeddings, and logical inferences and matrix operations. That is, the model behaves as if it is following a complex first-order reasoning process, but operates at the level of simple vectors and matrix representations.^30^ extended this to regularisation by addition of differentiable loss terms to the objective-based on propositionalisation of each first-order predicate. Guo *et al.*^56^ proposed a joint model, called KALE, which embeds facts from knowledge-graphs and logical rules, simultaneously. Here, the facts are represented as ground atoms with a calculated truth value in [0, 1] suggesting how likely that the fact holds. Logical rules (in grounded form) are then interpreted as complex formulae constructed by combining ground atoms with logical connectives, which are then modelled by fuzzy *t*-norm operators^57^. The truth value that results from this operation is nothing but a composition of the constituent ground atoms, allowing the facts from the knowledge graph to be incorporated into the model.

Li and Srikumar^58^ develop a method to constraint individual neural layers using soft logic based on massively available declarative rules in ConceptNet.^59^ incorporates first-order logic into low dimensional spaces by embedding graphs nodes and represents logical operators as learned geometric relations in the space.^60^ proposed ordering of embedding space based on rules mined from WordNet and found it to better prior knowledge and generalisation capabilities using these relational embeddings.^61^ show that domain-based regularisation in loss function can also help in constructing deep networks with less amount of data in prediction problems concerned with cloud computing. In^62^, a knowledge-based distant regularisation framework was proposed that utilises the distance information encoded in a knowledge-graph. It defines prior distributions of model parameters using knowledge-graph embeddings. They show that this results in an optimisation problem for a regularised factor analysis method.

### Semantic loss

Penalty terms can also be introduced on the extent to which the model’s prediction satisfies semantic domain constraints. For example, the domain may impose specific restrictions on the prediction (“output prediction must be in the range $3 \ldots 6$”). One way in which such information is provided is in the form of domain-constraints. Penalty terms are then introduced based on the number and importance of such constraints that are violated.

A recent work that is based on loss function is in^63^. Here the authors propose a semantic loss that signifies how well the outputs of the deep network matches some given constraints encoded as propositional rules. The general intuition behind this idea is that the semantic loss is proportional to a negative logarithm of the probability of generating a state that satisfies the constraint when sampling values according to some probability distribution. This kind of loss function is particularly useful for semi-supervised learning as these losses behave like self-information and are not constructed using explicit labels and can thus utilize unlabelled data.

^64^ proposed a framework to incorporate first-order logic rules with the help of an iterative distillation procedure that transfers the structured information of logic rules into the weights of neural networks. This is done via a modification to the knowledge-distillation loss proposed by Hinton et al.^65^. The authors show that taking this loss-based route of integrating rule-based domain-knowledge allows the flexibility of choosing a deep network architecture suitable for the intended task.

In^66^, authors construct a system for training a neural network with domain-knowledge encoded as logical constraints. Here the available constraints are transferred to a loss function. Specifically, each individual logic operation (such as negation, and, or, equality etc.) is translated to a loss term. The final formulation results in an optimisation problem. The authors extract constraints on inputs that capture certain kinds of convex sets and use them as optimisation constraints to make the optimisation tractable. In the developed system, it is also possible to pose queries on the model to find inputs that satisfy a set of constraints. In a similar line,^67^ proposed domain-adapted neural network (DANN) that works with a balanced loss function at the intersection of models based on purely domain-based loss or purely inductive loss. Specifically, they introduce a domain-loss term that requires a functional form of approximation and monotonicity constraints on the outputs of a deep network. Without detailing much on the underlying equations, it suffices to say that formulating the domain loss using these constraints enforces the model to learn not only from training data but also in accordance with certain accepted domain rules.

Another popular approach that treats domain knowledge as ‘domain constraints’ is semantic based regularisation^68,69^. It builds standard multilayered neural networks (e.g. MLP) with kernel machines at the input layer that deal with continuous-valued features. The output of the kernel machines is input to the higher layers implementing a fuzzy generalisation of the domain constraints that are represented in first-order logic. The regularisation term, consisting of a sum of fuzzy generalisation of constraints using *t*-norm operations, in the cumulative loss then penalises each violation of the constraints during the training of the deep network. In^70^ inject domain knowledge at training time via an approach that combines semantic based regularisation and constraint programming^71^. This approach uses the concept of ‘propagators’, which is inherent in constraint programming to identify infeasible assignments of variables to values in the domain of the variables. The role of semantic-based regularisation is to then penalise these infeasible assignments weighted by a penalty parameter. This is an example of constraints on inputs. In a similar line, In^72^ introduce domain-knowledge into a deep LSTM-based RNN at three different levels: constraining the inputs by designing a filtering module based on the domain-specific rules, constraining the output by enforcing an output range, and also by introducing a penalty term in the loss function.

A library for integrating symbolic domain-knowledge with deep neural networks was introduced recently in^73^. It provides some effective ways of specifying domain-knowledge, albeit restricted to (binary) hierarchical concepts only, for problems arising in the domain of natural language processing and some subset of computer vision. The principle of integration involves constraint satisfaction using a primal-dual formulation of the optimisation problem. That is: the goal is to satisfy the maximum number of domain constraints while also minimising the training loss, an approach similar to the idea proposed in^66,67,70^.

While adding a domain-based constraint term to the loss function may seem appealing, there are a few challenges. One challenge that is pointed out in a recent study^74^ is that incorporating domain-knowledge in this manner (that is: adding a domain-based loss to the standard problem-specific loss) may not always be suitable while dealing with safety-critical domains where 100% constraint satisfaction is desirable. One way to guarantee 100% domain-constraint satisfaction is by directly augmenting the output layer with some transformations and then deriving a new loss function due to these transformations. These transformations are such that they guarantee the output of the network to satisfy the domain constraints. In this study, called MultiplexNet^74^, the domain-knowledge is represented as a logical formula in disjunctive normal form (DNF) Here the output (or prediction) layer of a deep network is viewed as a multiplexor in a logical circuit that permits branching in logic. That is, the output of the network always satisfies one of the constraints specified in the domain knowledge (disjunctive formula).

The other form of semantic loss could be one that involves a human for post-hoc evaluation of a deep model constructed from a set of first-order rules. In this line, In^75^ proposed an analogical reasoning system intended for discovering rules by training a sequence-to-sequence model using a training set of rules represented in first-order logic. Here the role of domain-knowledge comes post training of the deep sequence model; that is, an evaluator (a human expert) tests each discovered rule from the model by unifying them against the (domain) knowledge base. The domain-knowledge here serves as some kind of a validation set where if the ratio of successful rule unification crosses a certain threshold, then the set of discovered rules are accepted.

## Transforming the model

**Figure 8:**
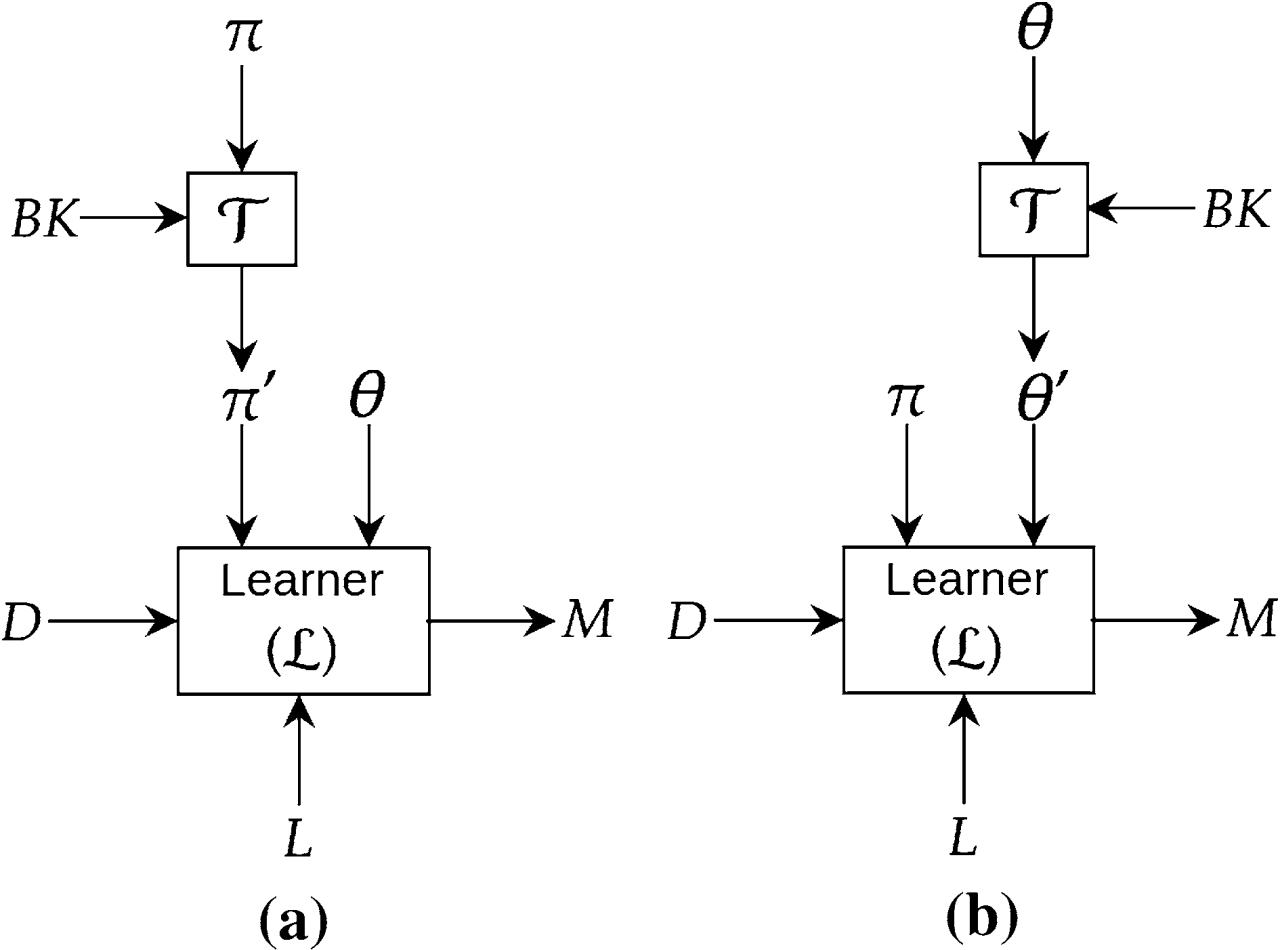
Introducing background knowledge into deep network by transforming the model (structure and parameter). In (**a**), the transformation block ${\mathcal {T}}$ takes a input structure of a model $\pi$ and outputs a transformed structure $\pi '$ based on background knowledge (*BK*). In (**b**), the transformation block ${\mathcal {T}}$ takes a set of parameters $\varvec{\theta }$ of a model and outputs a transformed set of parameters $\pi '$ based on background knowledge (*BK*).

Over the years, many studies have shown that domain knowledge can be incorporated into a deep network by introducing constraints on the model parameters (weights) or by making a design choice of its structure. Figure 8 shows a simple block diagram showing domain knowledge incorporation at the design stage of the deep network.

### Constraints on parameters

In a Bayesian formulation, there is an explicit mechanism for the inclusion of domain-knowledge through the use of priors. The regularisation terms in loss-functions, for example, can be seen as an encoding of such prior information, usually on the network’s structure. Priors can also be introduced on the parameters (weights) of a network. Explicitly, these would take the form of a prior distribution over the values of the weights in the network. The priors on networks and network weights represent our expectations about networks before receiving any data, and correspond to penalty terms or regularisers. Buntine and Weigend^76^ extensively study how Bayesian theory can be highly relevant to the problem of training feedforward neural networks. This work is explicitly concerned with choosing an appropriate network structure and size based on prior domain-knowledge and with selecting a prior for the weights.

The work by^77^ on Bayesian learning for neural networks also showed how domain-knowledge could help build a prior probability distribution over neural network parameters. In this, the shown priors allow networks to be “self-regularised” to not over-fit even when the complexity of the neural network is increased. In a similar spirit,^78^ showed how prior domain knowledge could be used to define ‘meta-features’ that can aid in defining the prior distribution of weights. These meta-features are additional information about each of the features in the available data. For instance, for an image recognition task, the meta-feature could be the relative position of a pixel (*x*, *y*) in the image. This meta information can be used to construct a prior over the weights for the original features.

#### Transfer learning

Transfer Learning is a mechanism to introduce priors on weights when data is scarce for a problem (usually called the “target” domain). Transfer learning relies on data availability for a problem similar to the target domain (usually called the “source” domain). From the position taken in this paper, domain-knowledge for transfer learning is used to change the structure or the parameter values (or both) for a model for the target problem. The nature of this domain-knowledge can be seen prior distributions on the structure and/or parameter-values (weights) of models for the target problem. The prior distributions for the target model are obtained from the models constructed for the source problem.

In practice, transfer learning from a source domain to a target domain usually involves a transfer of weights from models constructed for the source domain to the network in the target domain. This has been shown to boost performance significantly. From the Bayesian perspective, transfer learning allows the construction of the prior over the weights of a neural network for the target domain based on the posterior constructed in the source domain. Transfer learning is not limited by the kind of task (such as classification, regression, etc.) but rather by the availability of related problems. Language models are some of the very successful examples of the use of transfer learning, where the models are initially learnt on a huge corpus of data and fine-tuned for other more specialised tasks. In^79^ provides an in-depth review of some of the mechanisms and the strategies of transfer learning. Transfer learning need not be restricted to deep networks only: in a recent study,^80^ proposes a model that transfers knowledge from a neural network to a decision tree using knowledge distillation framework. The symbolic knowledge encoded in the decision tree could further be utilised for a variety of tasks.

A subcategory of transfer learning is one in which the problem (or task) remains the same, but there is a change in the distribution over the input data from the source and the target. This form of learning is viewed as an instance of domain-adaptation^34^. Similar to transfer learning, the knowledge is transferred from a source domain to a target domain in the form of a prior distribution over the model parameters. This form of domain-adaptation uses the same model structure as the source, along with an initial set of parameter values obtained from the source model. The parameter values are then fine-tuned using labelled and unlabelled data from the target data^81^. An example of this kind of learning is in^82^ where a BERT model is fine-tuned with data from multiple domains. There are some recent surveys along these lines:^83,84^.

### Specialised structures

DNN based methods arguably work best if the domain-knowledge is used to inspire their architecture choices^85^. There are reports on incorporating first-order logic constructs into the structure of the network. This allows neural-networks to operate directly on the logical sentences comprising domain-knowledge.

Domain-knowledge encoded as a set of propositional rules are used to constrain the structure of the neural network. Parameter-learning (updating of the network weights) then proceeds as normal, using the structure. The result could be thought of as learning weighted forms of the antecedents present in the rules. The most popular and oldest work along this line is Knowledge-Based Artificial Neural Network (KBANN)^13^ that incorporates knowledge into neural networks. In KBANN, the domain knowledge is represented as a set of hierarchically structured propositional rules that directly determines a fixed topological structure of a neural network^86^. KBANN was successful in many real-world applications; but, its representational power was bounded by pre-existing set of rules which restricted it to refine these existing rules rather than discovering new rules. A similar study is KBCNN^87^, which first identifies and links domain attributes and concepts consistent with initial domain knowledge. Further, KBCNN introduces additional hidden units into the network and most importantly, it allowed decoding of the learned rules from the network in symbolic form. However, both KBANN and KBCNN were not appropriate for learning new rules because of the way the initial structure was constructed using the initial domain knowledge base.

Some of the limitations described above could be overcome with the proposal of a hybrid system by Fletcher and Obradovic^88^. The system was able to learn a neural network structure that could construct new rules from an initial set of rules. Here, the domain knowledge is transformed into an initial network through an extended version of KBANN’s symbolic knowledge encoding. It performed incremental hidden unit generation thereby allowing construction or extension of initial rule-base. In a similar manner, there was a proposal for using Cascade ARTMAP^89^ which could not only construct a neural network structure from rules but also perform explicit cascading of rules and multistep inferencing. It was found that the rules extracted from Cascade ARTMAP are more accurate and much cleaner than the rules extracted from KBANN^90^.

In the late 1990s, Garcez and Zaverucha proposed a massively parallel computational model called CIL$^2$P based on feedforward neural network that integrates inductive learning from examples and domain knowledge, expressed as a propositional logic program^91^. A translation algorithm generates a neural network. Unlike KBANN, the approach uses the notion of “bipolar semi-linear” neurons. This allows the proof of a form of correctness, showing the existence of a neural-network structure that can compute the logical consequences of the domain-knowledge. The output of such a network, when combined into subsequent processing naturally incorporates the intended interpretation of the domain predicates. The authors extend this to the use of first-order logic programs: we have already considered this in Sect. 2.

A recent proposal focuses on embedding symbolic knowledge expressed as logical rules^91^. It considers two languages of representations: Conjunctive Normal Form (CNF) and decision-Deterministic Decomposable Negation Normal form (d-DNNF), which can naturally be represented as graph structures. The graph structures can be provided to a graph neural network (GNN) to learn an embedding suitable for further task-specific implementations.

Somewhat in a similar vein to the work by^29^, the work reported in^63^ considers as a set of propositional statements representing domain constraints. A deep network is then trained to find satisfying assignments for the constraints. Again, once such a network is constructed, it can clearly be used in subsequent processing, capturing the effect of the domain constraints. The network is trained using a semantic loss that we have described in Sect. 3.2.

In^58^ it is proposed to augment a language model that uses a deep net architecture with additional statements in first-order logic. Thus, given domain-knowledge encoded as first-order relations, connections are introduced into the network based on the logical constraints enforced by the domain-relations. The approach is related somewhat to the work in^92^ that does not explicitly consider the incorporation of domain-knowledge but does constrain a deep neural network’s structure by first grounding a set of weighted first-order definite clauses and then turning them into propositional programs.

We note that newer areas are emerging that use representations for domain-knowledge that go beyond first-order logic relations. This includes probabilistic first-order logic, as a way of including uncertain domain-knowledge^93^. One interesting way this is being used is to constrain the training of “neural predicates”, which represent probabilistic relations that are implemented by neural networks, and the framework can be trained in an end-to-end fashion^93,94^. In DeepProbLog^93^, for example, high-level logical reasoning can be combined with the sub-symbolic discriminative power of deep networks. For instance, a logic program for adding two digits and producing the output sum is straightforward. However, what if the inputs are images of the corresponding digits? Here, a deep network is used to map an image to a digit, while a (weighted) logic program, written in ProbLog^95^, for the addition operation is treated as the symbolic domain knowledge. The ProbLog program is extended with a set of ground neural predicates for which the weights correspond to the probability distribution of classes of digits (0 ...9). The parameters (weights of predicates and weights of neural network) are learned in an end-to-end fashion. A recent approach called DeepStochLog^94^ is a framework that extends the idea of neural predicates in DeepProbLog to definite clause grammars^96^. The reader may note that although DeepProbLog and DeepStochLog do not really transform the structure of the deep network, we are still considering these methods under the heading of specialised structures. This is because of the fact that the hybrid architecture is a tightly coupled approach combining probabilistic logic and deep neural networks.

One of the approaches involves transformation of a probabilistic logic program to graph-structured representation. For instance, in kLog^97^ the transformed representation is an undirected bipartite graph in the form of ‘Probabilistic Entity-Relationship model’^98^ which allows the use of a graph-kernel^99^ for data classification purpose, where each data instance is represented as a logic program constructed from data and background-knowledge. Another approach uses weighted logic programs or *templates* with GNNs^100^ demonstrating how simple relational logic programs can capture advanced graph convolution operations in a tightly integrated manner. However, it requires the use of a language of Lifted Relational Neural Networks (LRNNs)^101^.

An interesting proposal is to transform facts and rules, all represented in (weighted) first-order logic into matrix (or tensor) representations. Learning and inference can then be conducted on these matrices (or tensors)^102,103^ allowing faster computation. NeuralLog^104^, for example, extends this idea and constructs a multilayered neural network, to some extent, similar to the ones in LRNN consisting of fact layer, rule layer and literal layer etc. The learning here refers to the updates of the weights of the rules. Another work that translates domain-knowledge in first-order logic into a deep neural network architecture consisting of the input layer (grounded atoms), propositional layer, quantifier layer and output layer is^68^. Similar to LRNN, it uses the fuzzy *t*-norm operators for translating logical OR and AND operations.

Further emerging areas look forward to providing domain-knowledge as higher-order logic templates (or “meta-rules”: see^17^ for pointers to this area). To the best of our knowledge, there are, as yet, no reports in the literature on how such higher-order statements can be incorporated into deep networks.

**Table 1:** Some selected works, in no particular order, showing the principal approach of domain knowledge inclusion into deep neural networks. For each work referred here, we show the type of learner with following acronyms: Multilayer Perceptron (MLP), Convolutional Neural Network (CNN), Recurrent Neural Network (RNN), Graph Neural Network (GNN), Adaptive Resonance Theory-based Network Map (ARTMAP), DNN$^*$ refers to a DNN structure dependent on intended task. We use ‘MLP’ here to represent any neural network, that conforms to a layered-structure that may or maynot be fully-connected. RNN also refers to sequence models constructed using Long Short-Term Memory (LSTM) or Gated Recurrent Unit (GRU) cells.

Principal approach	Work (reference)	Type of learner
Transforming Data	DRM^24,25^	MLP
	CILP++^28^	MLP
	R-GCN^46^	GNN
	KGCN^61^	GNN
	KBRD^49^	GNN
	DG-RNN^44^	RNN
	DreamCoder^32^	DNN$^*$
	Gated-K-BERT^38^	Transformer
	VEGNN^5^	GNN
	BotGNN^6^	GNN
	KRISP^45^	GNN, Transformer
Transforming Loss	IPKFL^78^	CNN
	ILBKRME^30^	MLP
	HDNNLR^64^	CNN, RNN
	SBR^68^	MLP
	SBR^69^	CNN
	DL2^66^	CNN
	Semantic Loss^63^	CNN
	LENSR^91^	GNN
	DANN^67^	MLP
	PC-LSTM^72^	RNN
	DomiKnowS^73^	DNN*
	MultiplexNet^74^	MLP, CNN
	Analogy Model^75^	RNN
Transforming Model	KBANN^86^	MLP
	Cascade-ARTMAP^89^	ARTMAP
	CIL$^2$P^29^	RNN
	DeepProbLog^93^	CNN
	LRNN^101^	MLP
	TensorLog^103^	MLP
	Domain-Aware BERT^82^	Transformer
	NeuralLog^104^	MLP
	DeepStochLog^94^	DNN*

## Challenges and concluding remarks

We summarise our discussion on domain-knowledge as constraints in Table 1. We now outline some challenges in incorporating domain-knowledge encoded as logical or numerical constraints into a deep network. We first outline some immediate practical challenges concerning the logical constraints:

- There is no standard framework for translating logical constraints to neural networks. While there are simplification methods which first construct a representation of the logical constraint that a standard deep network can consume, this process has its limitations as described in the relevant section above.
- Logic is not differentiable. This does not allow using standard training of deep network using gradient-based methods in an end-to-end fashion. Propagating gradients via logic has now been looked at in^105^, but the solution is intractable and does not allow day-to-day use.
- Many neural network structures are directed acyclic graphs (DAGs). However, transforming logical formulae directly into neural network structures in the manner described in some of the discussed works can introduce cyclic dependencies, which may need a separate form of translations.

There are also practical challenges concerning the numerical constraints:

- We have seen that the numerical constraints are often provided with the help of modification to a loss function. Given some domain-knowledge in a logical representation, constructing a term in loss function is not straightforward.
- Incorporating domain-knowledge via domain-based loss may not be suitable for some safety-critical applications.
- The process of introducing a loss term often results in a difficult optimisation problem (sometimes constrained) to be solved. This may require additional mathematical tools for a solution that can be implemented practically.
- Deep network structures constrained via logical domain-knowledge may not always be scalable to large datasets.

It is possible to consider representing domain-knowledge not as logical or numeric constraints, but through statements in natural language. Recent rapid progress in the area of language models, for example, the models based on attention^106,107^ raises the possibility of incorporating domain-knowledge through conversations. While the precision of these formal representations may continue to be needed for the construction of scientific assistants, the flexibility of natural language may be especially useful in communicating commonsense knowledge to day-to-day machine assistants that need to an informal knowledge of the world^108,109^. Progress in this is being made (see, for example, https://allenai.org/aristo), but there is much more that needs to be done to make the language models required accessible to everyday machinery.

More broadly, incorporating domain-knowledge into learning is highlighted in^1^ as one of the Grand Challenges facing the foundations of AI and ML. Addressing this challenge effectively is seen as being relevant to issues arising in automated model-construction like data-efficiency and constraint-satisfaction. Additionally, it is suggested that developing a mapping of internal representations of the model to domain-concepts maybe necessary for acceptable explanations for the model’s predictions and for developing trust in the model.

It is now accepted that trust comes through understanding of how decisions are made by the machine-constructed models^110^, and what are the determining factors in these decisions. One important requirement of machine-constructed models in workflows with humans-in-the-loop is that the models are human-understandable. Domain-knowledge can be used in two different ways to assist this. First, it can constrain the kinds of models that are deemed understandable. Secondly, it can provide concepts that are meaningful for use in a model. Most of the work in this review has been focused on improving predictive performance. However, the role of domain-knowledge in constructing explanations for deep network models is also being explored (see, for example,^111^). However, that work only generates *post hoc* explanations that are locally consistent. Explanatory deep network models that identify true causal connections based on concepts provided as domain-knowledge remain elusive.

Domain-knowledge can also be used to correct biases^112^ built into a deep network either declaratively, through the use of constraints, or through the use of loss functions that include “ethical penalty” terms. Demonstrations of the use of domain-knowledge driven, ethics-sensitive machine learning have been available in the literature for some time^113^. Can these carry over to the construction of deep network models? This remains to be investigated.

The issues raised above all go beyond just the “how” questions related to the incorporation of domain-knowledge into deep neural networks. They provide pointers to why the use of domain-knowledge may extend beyond its utility for prediction.
